# Occurrence of hepatotoxicity with pazopanib and other anti-VEGF treatments for renal cell carcinoma: an observational study utilizing a distributed database network

**DOI:** 10.1007/s00280-016-3112-9

**Published:** 2016-07-20

**Authors:** Sumitra Shantakumar, Beth L. Nordstrom, Luc Djousse, Susan A. Hall, David R. Gagnon, Kathy H. Fraeman, Myrthe van Herk-Sukel, Karen Chagin, Jeanenne Nelson

**Affiliations:** 1 Worldwide Epidemiology, R&D, GlaxoSmithKline, 150 Beach Road, #26-00 Gateway West, Singapore, 189720 Singapore; 2 Worldwide Epidemiology Department, R&D, GlaxoSmithKline, Research Triangle Park, NC USA; 3 Center of Excellence in Epidemiology, Evidera, Lexington, MA USA; 4 Department of Veteran’s Affairs, Massachusetts Veterans Epidemiology Research and Information Center (MAVERIC), Boston VA Healthcare System, Boston, MA USA; 5 New England Research Institutes, Inc., Watertown, MA USA; 6 Department of Biostatistics, Boston University School of Public Health, Boston, MA USA; 7 PHARMO Institute for Drug Outcomes Research, Utrecht, The Netherlands; 8 Global Clinical Safety and Pharmacovigilance, GlaxoSmithKline, Collegeville, PA USA

**Keywords:** Hepatotoxicity, Liver chemistry, Renal cell carcinoma, Pazopanib, Anti-VEGF

## Abstract

**Purpose:**

To quantify the hepatic safety of pazopanib and comparator anti-vascular endothelial growth factor (VEGF) therapies in clinical practice among renal cell carcinoma (RCC) patients.

**Methods:**

A population-based cohort study of new anti-VEGF users was conducted in two US healthcare databases, Department of Veterans Affairs (VA) and an oncology practice network (Altos), and the PHARMO Database Network in The Netherlands. A common protocol was used to collect liver chemistry (LC) data from anti-VEGF initiation through 4 years of follow-up. In the VA population, suspected drug-induced liver injury (DILI) outcomes were investigated via chart review, with adjudication by hepatologists.

**Results:**

In Altos and VA, respectively, the total RCC patients were: pazopanib (156, 243), bevacizumab (122, 99), sorafenib (82, 249) and sunitinib (285, 751). PHARMO contained too few patients to be included. Few cases of alanine aminotransferase (ALT) ≥8× the upper limit of normal were seen across the anti-VEGF cohorts; incidence rates (per 100 person-years) ranged from 0 (sunitinib) to 8.2 (pazopanib) in Altos and from 0 (bevacizumab and sorafenib) to 2.1 (pazopanib) among VA patients. No cases of Hy’s law identified by combination LC elevations were seen in patients treated with pazopanib or bevacizumab; one case was observed in those treated with sorafenib, and two cases were found among sunitinib users. One case of adjudicated DILI was observed in a sunitinib-treated patient; none were found among patients treated with pazopanib, bevacizumab or sorafenib.

**Conclusions:**

Severe liver injury occurred infrequently during exposure to pazopanib and other anti-VEGF therapies in a population-based setting.

## Introduction

Pazopanib is a tyrosine kinase inhibitor indicated for the treatment of advanced renal cell carcinoma (RCC) and advanced soft tissue sarcoma. Prior to US Food and Drug Administration (FDA) approval of pazopanib for RCC in 2009, hepatotoxicity was observed in clinical trials, including two fatalities [1]. This led to a black box warning in the product label regarding liver safety and monitoring [2].

Pazopanib works in part by targeting tyrosine kinase inhibitors (TKIs) of vascular endothelial growth factor (VEGF) receptors. Other related medications indicated for RCC include the TKIs sunitinib and sorafenib as well as bevacizumab, an anti-VEGF monoclonal antibody [3]. Elevations in liver chemistry (LC) tests including alanine aminotransferase (ALT), aspartate aminotransferase (AST), alkaline phosphatase (ALP), and bilirubin have been found not only with pazopanib [1, 4, 5], but also in some studies with bevacizumab [6, 7], sunitinib [4, 8] and sorafenib [4, 9]. Similar to pazopanib, a boxed warning regarding hepatotoxicity exists for sunitinib [10]. As part of post-approval regulatory commitments for pazopanib, a post-marketing safety study was conducted in three electronic medical record (EMR) databases to examine the incidence of hepatotoxicity in RCC patients treated with pazopanib or comparator anti-VEGF drugs.

## Materials and methods

This population-based retrospective cohort study used a distributed data network that included data from three EMR databases: Altos, the US Department of Veterans Affairs Veterans Health Administration System (VA), and PHARMO Database Network. Altos contains EMR data for over 400,000 cancer patients seen in 150 outpatient oncology practices serving more than 1000 clinicians in the USA. Data obtained for each patient visit include diagnoses and treatment prescribed by or administered in the oncology clinic. Laboratory results are available through an automated link from the clinical laboratory. The VA is a national, integrated healthcare system with longitudinal clinical data for more than 12 million veterans during the most complete era of electronic data capture. Data on inpatient and outpatient encounters, procedures, all inpatient and outpatient medications, and all laboratory tests are available spanning 13 years. In addition, data on cause-specific mortality are available for all veterans. The Dutch-based PHARMO Institute has access to the PHARMO Database Network serving more than three million residents in The Netherlands. The database includes information on patient demographics, drug dispensing, hospital morbidity, clinical laboratory, pathology and general practitioner information.

Across the three databases, a common protocol was implemented by a coordinating center to ensure that patient selection and methodology were consistent within each database. New adult (18+ years) users of pazopanib, bevacizumab, sorafenib, or sunitinib with a diagnosis of RCC (ICD-9 codes 189.0 or 189.1) were identified from the earliest date of availability of pazopanib in the each health system (October 1, 2009, in the USA; February 1, 2011, in The Netherlands) through February 17, 2013 (6 weeks prior to the study end date of March 31, 2013). Because many patients beginning cancer treatment may be new to the oncology practice, no minimum duration of enrollment in the healthcare database was required prior to the drug start date. Included patients had at least one ALT and bilirubin measurement within 30 days before or on the drug start date to allow a comparison between drug initiation (baseline) and follow-up laboratory values. Patients prescribed more than one anti-VEGF agent concurrently were excluded, but patients could use more than one anti-VEGF agent during different time periods. Use of other antineoplastic treatments was permitted.

### Exposure to anti-VEGF drugs

Dates of exposure to the oral medications were derived from prescription records; when duration of treatment was not specified, a 30-day duration was assigned for pazopanib and sorafenib and 28 days for sunitinib. Bevacizumab was recorded as clinic administrations, with each administration assigned a duration of 14 days (i.e., the indicated interval between infusions). A continuous course of treatment was defined as sequential prescriptions for the index drug, including all refills indicated in the prescription, separated by gaps of no more than 30 days. A gap of greater than this duration flagged the end of one course of therapy and the start of a new treatment episode. Initiation of an alternate treatment for RCC that would not be expected to be used in combination with the index drug (including all of the other anti-VEGF drugs), with no repeat prescription of the initial drug after the start of the new treatment, was also used as an indicator of the end of a treatment episode.

### Baseline variables

Baseline is defined as the date of the anti-VEGF drug initiation (drug start date). The following baseline patient demographic, medical, and treatment characteristics were identified, as available in the various databases: age on the drug start date, gender, concomitant medications (defined as drugs ordered or administered within 30 days prior to the drug start date or during any period of anti-VEGF drug exposure), chemotherapy agents received prior to the drug start date, line of treatment of the qualifying regimen given on the drug start date (defined from all available information on chemotherapy exposure that preceded the regimen received on the drug start date) and presence and type of secondary tumors (identified using ICD-9 codes 196.xx–198.xx).

### Outcomes

The following LC elevation thresholds in relation to the upper limit of normal (ULN) were studied in all patients during the first exposure window to each anti-VEGF drug: ALT or AST (≥3x–4.99x, ≥5x–9.99x, ≥2x, ≥3x, ≥5x, ≥8x and ≥10x), ALP (>1x, >2x, >3x, ≥2x to 2.99x, ≥3x–4.99x and ≥5x), and total serum bilirubin (>1x, ≥1.5x–1.99x, ≥2x–4.99x, ≥2x and ≥5x). The combination of LC elevations indicative of possible Hy’s law was defined as ALT or AST ≥3x ULN, total bilirubin ≥2x ULN and ALP <2x ULN [11, 12].

Within the VA population only, drug-induced liver injury (DILI) events were also investigated. Patients were screened from the earliest to latest exposure date of any of the four anti-VEGF drugs, plus an additional 90 days past the last exposure date, for the occurrence of ALT >3x ULN concurrently with bilirubin >2x ULN, or for ICD-9 codes indicating potential DILI events (277.4, 570.x, 572.8, 573.3, 573.8, 576.8 or 782.4). An abstraction form was completed from the national EMR data for each patient who screened positive on either of these indicators; the specific anti-VEGF drugs received were blinded on the completed abstraction forms. Three members of a panel comprised by VA hepatologists and gastroenterologists independently reviewed each abstraction form and completed a case review form with a judgment on whether or not each case represented a DILI event. Following these independent reviews, cases were discussed and discrepancies resolved via teleconferences. Any disagreement that was not resolved through discussion was decided by majority vote. Case adjudication forms were then completed to reflect the final decision. An incident DILI event was deemed to have occurred if the expert panel agreed that there was at least a 50 % likelihood that the anti-VEGF drug contributed to a liver injury outcome.

### Statistical analysis

All analyses were run separately within each of the contributing databases. Data were analyzed at 6-month intervals during the first 4 years following FDA approval of pazopanib for treatment of RCC. At each interval, all cumulatively accrued patients were incorporated into the analyses; the present paper reports the findings from the final patient cohorts.

The baseline characteristics were summarized as number and percentage of patients for categorical variables or mean and standard deviation (SD) for continuous variables. Analysis of each category of LC elevation included only patients with no prior elevation at or above the lower threshold for the given elevation. For example, a patient with a baseline ALT of 3x ULN was ineligible for the analyses of ALT ≥3x ULN to 4.99x ULN, ≥2x ULN and ≥3x ULN, but was eligible for the other categories of ALT elevation. Cumulative incidence was calculated as the number of patients with an LC result in the given range divided by the number of patients evaluated. A one-sided, 97.5 % CI was estimated for cumulative incidence when the number of cases was zero. The incidence rate of each LC elevation was calculated as the number of patients with an elevation divided by the total amount of person-time. Total duration of follow-up was obtained by summing the total anti-VEGF drug exposure time across multiple separate periods of exposure for patients with gaps in treatment. Person-time was censored at the time of the first LC elevation for all incidence rates.

In addition to the overall analyses, two sets of stratified analyses were performed. First, the statistics described above were stratified by line of therapy on the drug start date; patients with the anti-VEGF drug as their first line of therapy were analyzed separately from those who received the drug as their second or higher line of therapy. Second, the time to event for patients with ALT ≥3x ULN, total bilirubin ≥2x ULN and for the Hy’s law combination were summarized. Ethical approvals/subject consent for the protection of human subjects via institutional review board or other ethical board review was obtained at the VA and PHARMO. Data obtained from the Altos database were deidentified and HIPPA compliant; thus, ethics approval was not needed.

## Results

A total of 156 pazopanib initiators in the Altos database and 243 in the VA qualified for the analysis. Pazopanib prescription in the PHARMO Database Network lagged considerably behind the US-based data sources, due in part to later reimbursement, formulary approval and availability of the drug in The Netherlands (February 2011), with only three pazopanib initiators identified by the end of the study period. This database was therefore excluded from the present analyses. Pazopanib was used for a mean duration of 4.2 months in Altos and 10.3 months in the VA population. The mean age was 65.1 years for Altos and 68.1 for the VA cohort; 64.2 and 98.3 % of patients were male in Altos and the VA, respectively (Table 1). Pazopanib was prescribed as first-line therapy for 38.5 % of Altos patients and 71.6 % of patients in the VA.

**Table 1:** Baseline characteristics of pazopanib and other anti-VEGF initiators among renal cell carcinoma patients in the Altos and Veterans Affairs (VA) databases

Patient characteristic, *n* (%)	Pazopanib		Bevacizumab		Sorafenib		Sunitinib	
	Altos	VA	Altos	VA	Altos	VA	Altos	VA
	*N* = 156	*N* = 243	*N* = 122	*N* = 99	*N* = 82	*N* = 249	*N* = 285	*N* = 751
Age, mean (SD)	65.7 (9.9)	67.5 (9.0)	63.5 (11.8)	67.3 (8.7)	66.5 (9.9)	66.8 (8.9)	67.0 (10.6)	67.1 (8.6)
Male gender	107 (68.6 %)	239 (98.4 %)	81 (66.4 %)	99 (100 %)	58 (70.7 %)	245 (98.4 %)	197 (69.1 %)	741 (98.7 %)
Metastases								
Lung	31 (19.9 %)	122 (50.2 %)	14 (11.5 %)	37 (37.4 %)	12 (15.6 %)	115 (46.2 %)	37 (13.0 %)	351 (46.7 %)
Liver	6 (3.8 %)	46 (18.9 %)	5 (4.1 %)	24 (24.2 %)	2 (2.4 %)	44 (17.7 %)	10 (3.5 %)	108 (14.4 %)
Bone or marrow	39 (25.0 %)	90 (37.0 %)	34 (27.9 %)	27 (27.3 %)	15 (18.3 %)	75 (30.1 %)	57 (20.0 %)	241 (32.1 %)
Brain or spinal cord	3 (1.9 %)	29 (11.9 %)	6 (4.9 %)	16 (16.2 %)	3 (3.7 %)	29 (11.7 %)	9 (3.2 %)	87 (11.6 %)
Adrenal gland	8 (5.1)	30 (12.4 %)	2 (1.6 %)	7 (7.1 %)	3 (3.7 %)	23 (9.2 %)	6 (2.1 %)	61 (8.1 %)
None	n/a	3 (1 %)	n/a	3 (3 %)	n/a	5 (2 %)	n/a	21 (2.8 %)
Line of treatment								
First line	60 (38.5 %)	174 (71.6 %)	37 (30.3 %)	59 (59.6 %)	26 (31.7 %)	186 (74.7 %)	233 (81.8 %)	558 (74.1 %)
Second line	40 (25.6 %)	50 (20.6 %)	43 (35.2 %)	20 (20.2 %)	28 (34.1 %)	51 (20.5)	40 (14.0 %)	69 (9.2 %)
Third line or higher	56 (35.9 %)	19 (7.8 %)	42 (34.4 %)	20 (20.2 %)	28 (34.2 %)	12 (4.8 %)	12 (4.2 %)	16 (2.1 %)

Overall, the number of patients with ALT ≥3x ULN ranged from 3 to 23 across the anti-VEGF cohorts, corresponding to an incidence rate of 6.5–25.9 per 100 person-years (py); the highest rates were observed among pazopanib-treated patients from the VA (Table 2). Few cases (range 0–4) of ALT ≥8x ULN were seen across the anti-VEGF cohorts, resulting in an incidence rate of 0–8.2 per 100 py. Rates of bilirubin elevation ≥2x ULN ranged from 2.6 to 11.9 per 100 py (range 1–30 cases), with the highest rates found among the patients from the VA treated with sorafenib or sunitinib. The median time to onset of these liver events varied substantially across the anti-VEGF cohorts (Table 3).

**Table 2:** Incidence rate (IR) per 100 person-years and cumulative incidence (%) of liver chemistry (LC) elevations among renal cell carcinoma patients in the Altos and Veterans Affairs (VA) populations

LC test and threshold	Pazopanib		Bevacizumab		Sorafenib		Sunitinib	
	Altos	VA	Altos	VA	Altos	VA	Altos	VA
ALT ≥2x ULN								
*N* cases/total	9/147	34/198	11/115	6/83	4/74	12/194	25/269	42/570
IR (95 % CI)	26.2 (22.1, 30.8)	41.5 (35.9, 47.7)	28.9 (23.8, 34.7)	16.6 (13.2, 20.6)	22.9 (18.0, 28.7)	15.3 (13.2, 17.6)	38.6 (34.1, 43.5)	16.0 (14.7, 17.3)
Cum. Inc. (%) (95 % CI)	6.1 (2.8, 11.3)	17.2 (12.2, 23.2)	9.6 (4.9, 16.5)	7.2 (2.7, 15.1)	5.4 (1.5, 13.3)	6.2 (3.2, 10.6)	9.3 (6.1, 13.4)	7.4 (5.4, 9.8)
ALT ≥3x ULN								
*N* cases/total	7/151	23/201	7/119	3/86	3/75	6/198	8/273	18/584
IR (95 % CI)	19.7 (16.7, 23.1)	25.9 (22.4, 29.7)	17.1 (14.2, 20.5)	7.7 (6.2, 9.5)	16.7 (13.1, 20.9)	7.2 (6.3, 8.3)	11.5 (10.2, 13.0)	6.5 (6.0, 7.1)
Cum. Inc. (%) (95 % CI)	4.6 (1.9, 9.3)	11.4 (7.4, 16.7)	5.9 (2.4, 11.7)	3.5 (0.7, 9.9)	4 (0.8, 11.3)	3.0 (1.1, 6.5)	2.9 (1.3, 5.7)	3.1 (1.8, 4.8)
ALT ≥8x ULN								
*N* cases/total	3/155	2/204	2/122	0/86	1/77	0/200	0/279	4/587
IR (95 % CI)	8.2 (6.9, 9.6)	2.1 (1.8, 2.4)	4.8 (4.0, 5.8)	0 (0, 11.1)	5.4 (4.3, 6.8)	0 (0, 5.2)	0 (0, 6.0)	1.4 (1.3, 1.5)
Cum. Inc. (%) (95 % CI)	1.9 (0.4, 5.6)	1.0 (0.1, 3.5)	1.6 (0.2, 5.8)	0 (0, 5.0)	1.3 (0.0, 7.0)	0 (0, 2.2)	0 (0, 1.6)	0.7 (0.2, 1.7)
Bilirubin >1x ULN								
*N* cases/total	14/152	31/198	8/117	18/86	6/79	27/190	23/268	104/579
IR (95 % CI)	40.3 (34.2, 47.3)	38.0 (32.9, 43.7)	21.5 (17.7, 25.7)	49.4 (39.5, 61.0)	33.7 (26.7, 42.0)	37.9 (32.7, 43.7)	33.8 (29.9, 38.1)	42.6 (39.2, 46.2)
Cum. Inc. (%) (95 % CI)	9.2 (5.1, 15.0)	15.7 (10.9, 21.5)	6.8 (3, 13.0)	20.9 (12.9, 31.1)	7.6 (2.8, 15.8)	14.2 (9.6, 20)	8.6 (5.5, 12.6)	18.1 (15, 21.4)
Bilirubin ≥1.5x ULN to 1.99x ULN								
*N* cases/total	3/155	11/203	2/119	3/88	2/81	11/202	6/277	31/590
IR (95 % CI)	8.1 (6.9, 9.5)	12.0 (10.4, 13.8)	5.2 (4.3, 6.2)	7.4 (5.9, 9.1)	10.5 (8.4, 13.1)	13.2 (11.4, 15.2)	8.4 (7.4, 9.4)	11.1 (10.3, 12.1)
Cum. Inc. (%) (95 % CI)	1.9 (0.4, 5.6)	5.4 (2.7, 9.5)	1.7 (0.2, 5.9)	3.4 (0.7, 9.6)	2.5 (0.3, 8.6)	5.4 (2.8, 9.5)	2.2 (0.8, 4.7)	5.3 (3.6, 7.4)
Bilirubin ≥2.0x ULN								
*N* cases/total	3/155	6/203	1/120	2/88	1/81	10/202	3/282	30/593
IR (95 % CI)	8.1 (6.8, 9.4)	6.5 (5.6, 7.5)	2.6 (2.1, 3.1)	4.9 (3.9, 6.0)	5.3 (4.2, 6.5)	11.9 (10.4, 13.7)	4.1 (3.6, 4.6)	10.5 (9.7, 11.4)
Cum. Inc. (%) (95 % CI)	1.9 (0.4, 5.6)	3.0 (1.1, 6.3)	0.8 (0.0, 4.6)	2.3 (0.3, 8.0)	1.2 (0.0, 6.7)	5.0 (2.4, 8.9)	1.1 (0.2, 3.1)	5.1 (3.4, 7.1)
Possible Hy’s law								
*N* cases/total	0/155	0/187	0/122	0/83	0/81	1/182	0/282	2/543
IR (95 % CI)	0 (0, 11.7)	0 (0, 9.6)	0 (0, 10.5)	0 (0, 17.1)	0 (0, 23.1)	1.3 (11.4, 15.2)	0 (0.6)	0.8 (4.2, 6.0)
Cum. Inc. (%) (95 % CI)	0 (0, 2.8)	0 (0, 4.1)	0 (0, 3.5)	0 (0, 6.7)	0 (0, 5.3)	0.6 (0.0, 3.0)	0 (0, 1.5)	0.4 (0.0, 1.3)

Persons with baseline elevations that exceeded a specified threshold were excluded from incidence analysis (hence, varying denominators). Possible Hy’s law: the combination of LC elevations indicative of possible Hy’s law was defined as ALT or AST ≥3x ULN, total bilirubin ≥2x ULN and ALP <2x ULN

A one-sided, 97.5 % CI was estimated for cumulative incidence when the number of cases was zero

*ALP* alkaline phosphatase, *ALT* alanine aminotransferase, *AST* aspartate aminotransferase, *CI* confidence interval, *Cum Inc* cumulative incidence, *SD* standard deviation, *ULN* upper limit of normal

**Table 3:** Time to select liver chemistry (LC) elevation events in Altos and Veteran Affairs databases

Drug	LC elevation	Altos		Veteran Affairs	
		No. events	Mean (median) days to event	No. events	Mean (median) days to event
Pazopanib	ALT ≥3x ULN	7	82.7 (61)	23	149 (56)
	Total bilirubin ≥2x ULN	3	73.3 (38)	6	311 (382)
	Possible Hy’s law	0	NA	0	NA
Bevacizumab	ALT ≥3x ULN	7	84.4 (84)	3	236 (218)
	Total bilirubin ≥2x ULN	1	112 (112)	2	97 (97)
	Possible Hy’s law	0	NA	0	NA
Sorafenib	ALT ≥3x ULN	3	57 (60)	6	160 (89)
	Total bilirubin ≥2x ULN	1	28 (28)	10	135 (57)
	Possible Hy’s law	0	NA	1	162 (162)
Sunitinib	ALT ≥3x ULN	8	79.1 (82.5)	18	96 (41)
	Total bilirubin ≥2x ULN	3	60.7 (77)	30	71 (41)
	Possible Hy’s law	0	NA	2	31 (31)

Possible Hy’s law: the combination of LC elevations indicative of possible Hy’s law was defined as ALT or AST ≥3x ULN, total bilirubin ≥2x ULN and ALP <2x ULN

*ALP* alkaline phosphatase, *ALT* alanine aminotransferase, *AST* aspartate aminotransferase, *ULN* upper limit of normal

 There was no discernible pattern in the variation of incidences for the first-line setting (Table 4) compared to the second-line setting (Table 5), aside from a drop in the incidence rate for most measures of ALT or bilirubin elevation among the pazopanib-treated cohort in both the Altos and VA databases. The exception was a greater incidence rate (per 100 py) of bilirubin ≥2x ULN in the second-line setting compared to the first-line setting, respectively, for pazopanib users in Altos (9.3, 6.3) and the VA (8.1, 5.4).

**Table 4:** Incidence rate (IR) per 100 person-years of liver chemistry (LC) elevation among renal cell carcinoma with selected treatment as first line of therapy, Altos and Veterans Affairs (VA)

LC test and threshold	Pazopanib		Bevacizumab		Sorafenib		Sunitinib	
	Altos	VA	Altos	VA	Altos	VA	Altos	VA
ALT ≥2x ULN								
*N* cases/total	3/57	24/123	2/35	2/48	1/24	12/136	24/221	37/500
IR (95 % CI)	20.8 (15.8, 27)	50.7 (42.2, 60.5)	13.4 (9.3, 18.6)	9.0 (6.6, 11.9)	13.4 (8.6, 19.9)	19.9 (16.7, 23.5)	44.2 (38.6, 50.5)	15.4 (14.0, 16.8)
ALT ≥3x ULN								
*N* cases/total	4/59	20/125	2/36	2/49	1/24	6/139	7/224	16/513
IR (95 % CI)	27.2 (20.7, 35.0)	39.4 (32.8, 46.9)	13.3 (9.3, 18.4)	8.6 (6.4, 11.4)	13.3 (8.5, 19.8)	9.2 (7.8, 10.9)	11.9 (10.4, 13.6)	6.3 (5.8, 6.9)
ALT ≥8x ULN								
*N* cases/total	2/60	2/127	1/37	0/49	0/24	0/140	0/230	4/516
IR (95 % CI)	13.0 (9.9, 16.8)	3.4 (2.9, 4.1)	6.6 (4.7, 9.1)	0 (0, 18.7)	0 (0, 57.3)	0 (0, 6.6)	0 (0, 7.0)	1.6 (1.4, 1.7)
Bilirubin >1x ULN								
*N* cases/total	8/59	20/122	2/37	12/51	2/25	21/132	17/219	97/508
IR (95 % CI)	54.1 (41.2, 69.7)	39.7 (33.0, 47.4)	13.4 (9.4, 18.5)	54.4 (40.5, 71.5)	29.0 (18.7, 42.8)	38.1 (31.9, 45.2)	29.3 (25.5, 33.4)	43.7 (40.0, 47.6)
Bilirubin ≥1.5x ULN to 1.99x ULN								
*N* cases/total	2/60	8/126	1/37	2/51	0/26	10/142	5/226	29/520
IR (95 % CI)	12.6 (9.6, 16.2)	14.7 (12.3, 17.5)	6.6 (4.7, 9.1)	8.1 (6, 10.6)	0 (0, 55.8)	15.3 (12.9, 18.1)	8.3 (7.2, 9.4)	11.4 (10.4, 12.4)
Bilirubin ≥2x ULN								
*N* cases/total	1/60	3/126	1/37	1/51	0/26	9/142	3/231	26/523
IR (95 % CI)	6.3 (4.8, 8.1)	5.4 (4.5, 6.5)	6.6 (4.6, 9.1)	4.0 (3.0, 5.2)	0 (0, 55.8)	13.7 (11.5, 16.1)	4.8 (4.2, 5.5)	10.0 (9.1, 10.8)
Possible Hy’s law								
*N* cases/total	0/60	0/115	0/37	0/47	0/25	1/126	0/231	2/477
IR (95 % CI)	0 (0, 27.1)	0 (0, 24.9)	0 (0, 28.6)	0 (0, 24.9)	0 (0, 56.3)	1.6 (1.4, 1.9)	0 (0, 7.0)	0.8 (4.5, 6.7)

Possible Hy’s law: the combination of LC elevations indicative of possible Hy’s law was defined as ALT or AST ≥3x ULN, total bilirubin ≥2x ULN and ALP <2x ULN

*ALP* alkaline phosphatase, *ALT* alanine aminotransferase, *AST* aspartate aminotransferase, *CI* confidence interval, *SD* standard deviation, *ULN* upper limit of normal

**Table 5:** Incidence rate (IR) per 100 person-years of liver chemistry (LC) elevation among renal cell carcinoma with selected treatment as second line of therapy or higher, Altos and Veterans Affairs (VA)

LC test and threshold	Pazopanib		Bevacizumab		Sorafenib		Sunitinib	
	Altos	VA	Altos	VA	Altos	VA	Altos	VA
ALT ≥2x ULN								
*N* cases/total	6/90	10/75	9/80	4/35	3/50	0/58	1/48	5/70
IR (95 % CI)	30.0 (24.1, 36.9)	28.9 (22.7, 36.2)	38.9 (30.8, 48.4)	28.8 (20.1, 40.1)	30.0 (22.3, 39.5)	0 (0, 24.2)	9.5 (7.0, 12.6)	22.4 (17.5, 28.3)
ALT ≥3x ULN								
*N* cases/total	3/92	3/76	5/83	1/37	2/51	0/59	1/49	2/71
IR (95 % CI)	14.4 (11.6, 17.7)	7.9 (6.2, 9.9)	19.4 (15.4, 24.0)	6.3 (4.5, 8.7)	19.0 (14.2, 25.0)	0 (0, 24.2)	9.4 (6.9, 12.4)	8.1 (6.3, 10.2)
ALT ≥8x ULN								
*N* cases/total	1/95	0/77	1/85	0/37	1/53	0/60	0/49	0/71
IR (95 % CI)	4.7 (3.8, 5.7)	0 (0, 11.4)	3.8 (3.0, 4.7)	0 (0, 27.5)	9.2 (6.9, 12.1)	0 (0, 23.8)	0 (0, 41.1)	0 (0, 17.3)
Bilirubin >1x ULN								
*N* cases/total	6/93	11/76	6/80	6/35	4/54	6/58	6/49	7/68
IR (95 % CI)	30.1 (24.3, 36.9)	35.2 (27.8, 44.1)	26.8 (21.3, 33.4)	41.8 (29.1, 58.1)	36.6 (27.5, 47.8)	37.2 (28.2, 48.1)	59.9 (44.3, 79.1)	31.7 (24.6, 40.2)
Bilirubin ≥1.5x ULN to 1.99x ULN								
*N* cases/total	1/95	3/77	1/82	1/37	2/55	1/60	1/51	2/70
IR (95 % CI)	4.7 (3.8, 5.8)	8.0 (6.3, 10.0)	4.2 (3.4, 5.3)	6.3 (4.4, 8.7)	18.0 (13.5, 23.3)	5.5 (4.2, 7.1)	8.7 (6.5, 11.5)	8.4 (6.6, 10.7)
Bilirubin ≥2x ULN								
*N* cases/total	2/95	3/77	0/83	1/37	1/55	1/60	0/51	4/70
IR (95 % CI)	9.3 (7.6, 11.4)	8.1 (6.4, 10.2)	0 (0, 18.3)	6.3 (4.4, 8.7)	9.0 (6.8, 11.7)	5.6 (4.3, 7.2)	0 (0, 38.3)	17.0 (13.2, 21.4)
Possible Hy’s law								
*N* cases/total	0/95	0/72	0/85	0/36	0/56	0/56	0/51	0/66
IR (95 % CI)	0 (0, 20.4)	0 (0, 40.4)	0 (0, 16.6)	0 (0, 54.1)	0 (0, 39.1)	0 (0, 30.9)	0 (38.2)	0 (0, 36.5)

Possible Hy’s law: the combination of LC elevations indicative of possible Hy’s law was defined as ALT or AST ≥3x ULN, total bilirubin ≥2x ULN and ALP <2x ULN

*ALP* alkaline phosphatase, *ALT* alanine aminotransferase, *AST* aspartate aminotransferase, *CI* confidence interval, *SD* standard deviation, *ULN* upper limit of normal

No cases of Hy’s law identified by combination LC elevations were seen in patients treated with pazopanib or bevacizumab; three cases were observed in those treated with sorafenib (*n* = 1) and sunitinib (*n* = 2) at the VA. Among the VA population, one case of adjudicated DILI, as captured by ICD-9 codes, was found in a sunitinib-treated patient. No adjudicated case of DILI was seen among patients treated with pazopanib, bevacizumab or sorafenib.

## Discussion

Results from this study show that severe liver injury occurred infrequently during pazopanib exposure in a population-based, real-world setting. Further, no combinations of LC elevations meeting the laboratory definition of Hy’s law were observed in all pazopanib-exposed patients from either database, and no pazopanib-related adjudicated DILI outcomes were identified among more than 300 RCC patients accrued in the VA. Few liver toxicity events were identified among patients using the other anti-VEGF agents.

Liver toxicity among pazopanib users was less frequently reported in the present population-based study compared to a recent report of 1149 pazopanib-treated RCC patients from nine prospective clinical trials [1]. Among the trial participants, 23 % of patients developed elevated ALT >3x ULN compared to 4.6 % of patients in the Altos population and 11.4 % among VA patients for ALT ≥3x ULN. Further, the cumulative incidence of severe liver toxic was higher among clinical trial patients (ALT >8x ULN, 8.4 %) compared to the Altos (1.9 %) and VA (1.0 %) population (ALT ≥8x ULN). It is possible that transient or asymptomatic LC elevations were missed in the present study of real-world patients where liver monitoring may be less rigorous compared to a trial setting.

Several factors are potential limitations in this study. Small numbers of patients in several analytic groups, including line of therapy, led to some imprecise estimates. Advanced RCC could not be identified separately from total kidney cancers; metastases may be underreported, especially in the Altos database. Exposure time to oral medications, including pazopanib, imputed from dates for orders and refills may be imprecise, resulting in inaccurate computation of person-time used in the incidence rate calculations. It is also possible that some pazopanib users were not new users on the drug start date, if prior pazopanib prescriptions did not appear in the database, leading to an underestimate for the rate of LC elevations during treatment. In the Altos population, serious liver events resulting in hospitalization and LC elevations measured outside the oncology practice EMR could not be captured, unless the information was manually entered into the EMR database, thus potentially underestimating the rate of severe LC elevations. The VA database included hospital data and can therefore allow for fuller capture of patient experiences, although patients who received medical care outside of the VA system may also have had missing LC test results. It is possible the ICD-9 codes used for screening potential cases of DILI in the VA population may be inaccurate.

This population-based study has a number of strengths. Patients come from real-world oncology settings, allowing for more generalizable results compared to clinical trial populations. The EMR contains data from usual clinical practice, with issues such as dosing and adherence uninfluenced by a trial protocol. EMRs provide longitudinal data allowing for better capture of events compared to reliance on individual spontaneous adverse event reports. The study period duration of clinical trials is generally short compared to the present study with more extensive follow-up time for observing LC elevations. This study allows for comparison of LC elevations among a large number of patients with the same indication across different comparator drugs and multiple healthcare databases with diverse patient populations. The chart review and adjudication process by a panel of three physicians allowed for careful assessment of suspected DILI events in the VA population; this approach represents a gold standard of outcome ascertainment in retrospective studies.

Current post-marketing surveillance of pazopanib and other anti-VEGF drugs in RCC patients showed a relatively low frequency of serious liver events for approximately 4 years of data collection. More data are needed to estimate the rates of DILI or liver failure in pazopanib users. Understanding the incidence of severe liver injuries in RCC patients newly using pazopanib and other marketed anti-VEGF agents in real-world clinical practice helps inform patients’ hepatic safety profile.
